# The Roles of Probiotics in the Gut Microbiota Composition and Metabolic Outcomes in Asymptomatic Post-Gestational Diabetes Women: A Randomized Controlled Trial

**DOI:** 10.3390/nu14183878

**Published:** 2022-09-19

**Authors:** Zubaidah Hasain, Raja Affendi Raja Ali, Hajar Fauzan Ahmad, Ummul Fahri Abdul Rauf, Seok Fang Oon, Norfilza Mohd Mokhtar

**Affiliations:** 1 Department of Physiology, Faculty of Medicine, Universiti Kebangsaan Malaysia, Kuala Lumpur 56000, Malaysia; 2 Unit of Physiology, Faculty of Medicine & Defence Health, Universiti Pertahanan Nasional Malaysia, Kuala Lumpur 57000, Malaysia; 3 Gastroenterology Unit, Department of Medicine, Faculty of Medicine, Universiti Kebangsaan Malaysia, Kuala Lumpur 56000, Malaysia; 4 GUT Research Group, Faculty of Medicine, Universiti Kebangsaan Malaysia, Kuala Lumpur 56000, Malaysia; 5 Department of Industrial Biotechnology, Faculty of Industrial Sciences and Technology, Universiti Malaysia Pahang, Gambang 26300, Malaysia; 6 Center for Research in Advanced Tropical Bioscience, Universiti Malaysia Pahang, Gambang 26300, Malaysia; 7 Department of Mathemathics, Centre for Foundation Studies, Universiti Pertahanan Nasional Malaysia, Kuala Lumpur 56000, Malaysia; 8 B-Crobes Laboratory Sdn. Bhd., Perak 31400, Malaysia

**Keywords:** gestational diabetes, gut microbiota, insulin resistance, probiotic, glycemic control, inflammation

## Abstract

Probiotics are widely used as an adjuvant therapy in various diseases. Nonetheless, it is uncertain how they affect the gut microbiota composition and metabolic and inflammatory outcomes in women who have recently experienced gestational diabetes mellitus (post-GDM). A randomized, double-blind, placebo-controlled clinical trial involving 132 asymptomatic post-GDM women was conducted to close this gap (Clinical Trial Registration: NCT05273073). The intervention (probiotics) group received a cocktail of six probiotic strains from *Bifidobacterium* and *Lactobacillus* for 12 weeks, while the placebo group received an identical sachet devoid of living microorganisms. Anthropometric measurements, biochemical analyses, and 16S rRNA gene sequencing results were evaluated pre- and post-intervention. After the 12-week intervention, the probiotics group’s fasting blood glucose level significantly decreased (mean difference −0.20 mmol/L; *p* = 0.0021). The HbA1c, total cholesterol, triglycerides, and high-sensitivity C-reactive protein levels were significantly different between the two groups (*p* < 0.05). Sequencing data also demonstrated a large rise in the *Bifidobacterium adolescentis* following probiotic supplementation. Our findings suggest that multi-strain probiotics are beneficial for improved metabolic and inflammatory outcomes in post-GDM women by modulating gut dysbiosis. This study emphasizes the necessity for a comprehensive strategy for postpartum treatment that includes probiotics to protect post-GDM women from developing glucose intolerance.

## 1. Introduction

Gestational diabetes mellitus (GDM) is defined as an episode of high blood glucose levels detected during the second or third trimester in pregnant women without overt diabetes [1]. Bellamy et al. [2] reported that women who have had GDM in the past (post-GDM) have a sevenfold greater risk of progressing to type 2 diabetes mellitus (T2DM) compared with normoglycemic women. Moreover, a recent meta-analysis of 20 studies found that post-GDM women have a nearly 10-fold higher risk of developing T2DM over their lifetimes than healthy women [3]. The main risk factors were also noted by the authors, and these include Asian ethnicity, obesity, advanced maternal age, multiparity, familial history of T2DM, early GDM diagnosis, recurring history of GDM, and the need for hypoglycemic drugs during pregnancy [4,5]. Urbanization, unhealthy diet, a sedentary lifestyle, and poor compliance with postpartum screening were also added by other authors to this list [3,6]. These findings highlight the necessity for a postpartum preventive care strategy that is well-structured to slow the progression of T2DM in women with the aforementioned risk factors and lessen the financial burden of health care.

The American Diabetes Association recommends that post-GDM women lead healthy lifestyles (diet and exercise) and take metformin if they are prediabetic [7], as these strategies have been shown to reduce the risk of developing diabetes by 53% and 50%, respectively, in post-GDM women [8]. This advice is dubious, though, as many women fail to comply with the recommended lifestyle modifications [9,10,11], and it may not always be necessary to take medication [12]. The risks and advantages of prescribing metformin to asymptomatic post-GDM women should be considered before doing so. Metformin is associated with a few of the mild to serious side effects, including hypoglycemia, gastrointestinal disturbances, and dizziness [13,14,15]. An alternative therapeutic strategy that can safely and effectively improve glucose homeostasis in asymptomatic post-GDM women is therefore required.

According to the available evidence, aberrant alterations in the normal composition of the gut microbiota (gut dysbiosis) in pregnant women with GDM are directly associated to alterations in metabolism and may persist into the postpartum period [16,17,18]. Specifically, in pregnant women with GDM, *Sutterella* is positively correlated with C-reactive protein levels, while *Collinsella* has a strong direct relationship with insulin/homeostasis model assessment of insulin resistance (HOMA-IR) levels [18]. However, the connection between gut dysbiosis and glucose intolerance (GI) in post-GDM women during the postpartum period has not been extensively researched, and the information that is available data is contradictory. For example, Fugmann et al. [19] showed that post-GDM women had a lower abundance of Firmicutes than in healthy normoglycemic women did [20]. Contrarily, Hasan et al. [21] found no appreciable distinction between the gut microbial profiles of women with and without GDM five years after delivery. In post-GDM women, it is hypothesized that food consumption, gut microbiota composition, lipopolysaccharide (LPS), and short-chain fatty acids (SCFAs) imbalance glucose homeostasis by causing inflammatory response and intestinal permeability [20,22]. Similar observations in diabetic adults support the idea that managing gut dysbiosis and inflammatory remodeling can be a successful preventive measure for managing GI in post-GDM women [23].

Recent studies indicate that probiotics influence host metabolism favorably in a variety of diseases via modifying the gut microbiota and inflammatory response [24,25,26,27,28,29]. In a consensus statement, the International Scientific Association for Probiotic and Prebiotic has defined probiotics as “living microorganisms that, when administered in adequate amounts, impart a health benefit on the host” [30]. Probiotics can be consumed during pregnancy and after delivery because they are generally safe, well-tolerated, and they have a high compliance rate [31,32]. Probiotic supplementation has been proven to be beneficial for controlling fasting blood glucose (FBG), fasting serum insulin (FSI), and HOMA-IR levels [32,33,34]. Moreover, high-sensitivity C-reactive protein (hs-CRP), interleukin-6 (IL-6), and tumor necrosis-α (TNF-α) levels have all been shown to be reduced with probiotic administration [32,34]. Similar positive effects of probiotics have been found in adults with prediabetes and T2DM [35,36,37].

To the best of our knowledge, data on probiotic supplementation in post-GDM women who are asymptomatic is still lacking and inconsistent. We designed a randomized controlled trial to look at how probiotics affected the metabolic and inflammatory outcomes in asymptomatic post-GDM women in order to close this knowledge gap in the literature. With the assumption that probiotic supplementation may have some positive impacts on reducing gut dysbiosis and enhancing metabolic and inflammatory outcomes in asymptomatic post-GDM women, the objective of this study was to ascertain the function of probiotic supplementation in gut microbial profiles.

## 2. Materials and Methods

### 2.1. Study Design

A 12-week randomized, double-blind, placebo-controlled, parallel-group clinical trial was conducted by the Faculty of Medicine, Universiti Kebangsaan Malaysia (UKM) (Cheras, Malaysia). The allocation ratio was 1:1. The design and reporting of this trial were in accordance with CONSORT 2010 guidelines [38]. The initial protocol aimed to recruit only post-GDM women with postpartum GI and asymptomatic of hyperglycemia. To improve our recruitment process, we expanded the eligibility criteria to include post-GDM women who were overweight/obese but had normal postpartum glucose tolerance. This criterion was added because obesity is also the primary risk factor for persistent postpartum GI.

### 2.2. Participant Recruitment

The participant’s eligibility was evaluated between four and eight weeks after delivery. Eligible participants were women aged 18–45 years who had attended postnatal follow-up at UKM Medical Center with a recent history of GDM, were willing to participate in the study, and had either postpartum GI, overweight (body mass index (BMI) ≥ 23 kg/m^2^), or obesity (BMI ≥ 27.5 kg/m^2^) during eligibility assessment. Postpartum GI was diagnosed using a 2 h, 75 g oral glucose tolerance test (OGTT), and the condition was then classified in accordance with Malaysian clinical practice guidelines [39]. Postpartum GI diagnosis includes:

(1) Impaired fasting glucose (IGT), FBG level ≥ 6.1 mmol/L and 2-h post-prandial (2HPP) level < 7.8 mmol/L.
(2) Impaired glucose tolerance (IGT), FBG level < 6.1 mmol/L and 2HPP level ≥ 7.8 mmol/L.
(3) Combination of IFG and IGT, FBG level between 6.1–6.9 mmol/L and 2HPP level between 7.8–11.0 mmol/L.

Women with post-GDM who required hypoglycemic medication upon postpartum GI diagnosis and had postpartum FBG levels > 7.0 mmol/L, 2HPP glucose levels > 11.1 mmol/L were excluded. Further exclusion criteria were women with post-GDM who had underlying medical illnesses that required ongoing treatments (such as pre-pregnancy diabetes, hypertension, congestive heart failure, renal failure, liver cirrhosis, gastrointestinal diseases, autoimmune diseases, and cancer) at the time of recruitment or during the intervention. Additionally, before and during the intervention, participants who had taken antibiotics or regularly consumed foods or supplements rich in prebiotics/probiotics, called synbiotics, were disqualified. Prior to recruitment, there was a minimum 12-week washout period for antibiotics, prebiotics, probiotics, and synbiotics.

The trial was performed for three years (between 2018 and 2021) in the Department of Physiology, Faculty of Medicine, UKM, Malaysia. Participants’ baseline characteristics, such as sociodemographic, antepartum, and postpartum information, were recorded.

### 2.3. Intervention and Compliance

The probiotics and placebo groups were created at random from among all eligible participants. The intervention was started later, between three and six months postpartum, to reduce the impacts of the tight dietary restrictions among Asian women during the early confinement period [40]. The intervention lasted 12 weeks. The participants in the probiotics group received HEXBIO^®^ Microbial Cell Preparation (MCP), from B-Crobes Laboratory Sdn. Bhd, which contains MCP^®^ BCMC^®^ strains. It was made into a white granular powder with an orange flavor that was packaged in an aluminum foil sachet. Each sachet of total 30 billion colony-forming units (CFU) contains the six probiotic strains (*Lactobacillus acidophilus* BCMC^®^ 12130 (107 mg), *Lactobacillus casei* subsp. BCMC^®^ 12313 (107 mg), *Lactobacillus lactis* BCMC^®^ 12451 (107 mg), *Bifidobacterium bifidum* BCMC^®^ 02290 (107 mg), *Bifidobacterium infantis* BCMC^®^ 02129 (107 mg), and *Bifidobacterium longum* BCMC^®^ 02120 (107 mg)). Meanwhile, participants in the placebo group received an identical sachet with non-microbial material.

Before each meal, the participants were instructed to consume one sachet twice daily (in the morning and evening), which they were to mix with a glass of water. It was made clear to participants that they should refrain from eating or drinking anything hot for two hours before and after ingesting the interventional product. The remaining sachets were stored in a dry place that was kept away from direct sunlight at or below 25 °C.

Reminders were sent to the participants on a regular basis through phone messages to ensure that they were following instructions for using the interventional products and to monitor any side effects. Participants with mild side effects were encouraged to continue the program. Participants who developed severe adverse effects, however, were removed from the intervention, but they were advised to complete post-intervention assessments. Remaining sachets were counted after the 12-week intervention to estimate the compliance rate. Between 85% and 100% of the required compliance was deemed acceptable [41].

### 2.4. Dietary Intake and Physical Activity Assessments

Participants’ baseline dietary intake and physical activities were assessed before the intervention. A three-day food record made up of two weekdays and one weekend was evaluated to estimate participants’ macronutrient intake. The participant’s physical activity was evaluated using the short questionnaire to assess health-enhancing activity (SQUASH). Participants were to estimate the number of days and hours spent doing each of four habitual activity domains (commuting, leisure, household, and work) during the course of an ordinary week in the past month. The total weekly minutes for each domain were added up to total the participants’ estimated overall physical activity.

The advice given to participants was to maintain their same lifestyle and refrain from making sudden changes to their routine dietary intake and physical activity. They were also told to stay away from foods or supplements that are rich in prebiotics, probiotics, or synbiotics. Dietary intake and physical activity assessments were repeated at the end of the intervention to monitor the changes in food intake and physical activity during the intervention.

### 2.5. Outcomes Measurements

All outcomes measurements were taken before the intervention and repeated after the trial (after a 12-week intervention). The primary outcomes were the mean differences between baseline and 12-week intervention in FBG levels and other glycemic control biomarkers. The secondary outcomes were the mean differences in anthropometric measurements, blood pressure readings, lipid biomarkers, inflammatory biomarkers, and changes in gut microbial profiles during the course of the 12-week intervention.

#### 2.5.1. Anthropometric and Blood Pressure Measurements

Information on height was retrieved from the antenatal record book, while pre-pregnancy body weight was self-reported. Following a previous publication, anthropometric measurements such as postpartum body weight, BMI, and waist circumference (WC) were measured before and after the intervention [20]. A digital blood pressure monitor (Omron HEM-7120; Omron Healthcare Europe BV, Hoofddorp, The Netherlands) was used to take blood pressure readings while the subject was seated. After at least 10 min of rest, the measurement was performed twice, and the mean of the two readings was calculated.

#### 2.5.2. Biochemical Analysis

Approximately 10 to 15 mL of blood was collected following a 10 h fast before and after the intervention. Among the indicators for glycemic control were FBG, HbA1c, FSI, fasting serum active glucagon-like peptide-1 (GLP-1), and HOMA-IR. FBG levels were measured using the hexokinase method on a Siemens ADVIA 2400 analyzer (Siemens Healthcare Diagnostics, Tarrytown, NY, USA), and HbA1c levels were determined using turbidimetric inhibition immunoassay on a Roche Cobas 513 analyzer (Roche Diagnostics GmbH, Mannheim, Germany). FSI and active GLP-1 levels were analyzed using EMD Millipore’s MILLIPLEX^®^ MAP Human Metabolic Hormone Magnetic Bead Panel based on the Luminex^®^ xMAP^®^ technology (Merck KGaA, Darmstadt, Germany). A previous publication served as the basis for calculating HOMA-IR levels [20].

Total cholesterol levels were determined using the enzymatic method and followed by a Trinder endpoint. Trinder endpoint was used after measuring the triglyceride (TG) levels using the Fossatti three-step enzymatic reaction. Both lipid biomarkers were analyzed on a Siemens ADVIA 2400 analyzer (Siemens Healthcare Diagnostics, Tarrytown, NY, USA). Inflammatory biomarkers include hs-CRP, IL-1β, IL-6, and IL-8. The hs-CRP levels were measured using the latex-enhanced immunoturbidimetric assay method on the Siemens ADVIA 2400 analyzer (Siemens Healthcare Diagnostics, Tarrytown, NY, USA). Serum IL-1β, IL-6, and IL-8 levels were analyzed using EMD Millipore’s MILLIPLEX^®^ MAP Human High Sensitivity T Cell Panel based on the Luminex^®^ xMAP^®^ technology (Merck KGaA, Darmstadt, Germany).

#### 2.5.3. Gut Microbial Analysis

The gut microbial profiles of probiotics and placebo groups were studied before and after the intervention. Detailed descriptions of the gut microbial analysis were given in an earlier work [20]. In brief, 24 early participants (12 participants per group) had their feces collected before and after the intervention. Total bacterial genome DNA was extracted using the Fast DNA™ SPIN Kit for soil (MP Biomedical, Santa Ana, CA, USA). Additional analyses were carried out, such as the library preparation based on the amplification of V3-V4 regions of 16S rRNA using the NEBNext^®^ Ultra^TM^ DNA Library Prep Kit for Illumina (New England Bio Labs, Ipswich, MA, USA), high-throughput next-generation sequencing using Illumina HiSeq2500 platform (Illumina, Inc., San Diego, CA, USA), and bioinformatics analysis for microbial genomic analysis. As part of an open-source workflow provided by MOTHUR software, the representative sequence of the OTUs screening and species annotation alignment for each taxonomic rank were performed by referring to the SILVA-based bacterial reference database (Silva release 128) [42].

The top 10 relative abundances of gut microbial taxa at the phylum level for each participant were selected and presented as stacked bars using GraphPad Prism. The sequence data were processed in QIIME (Version 1.7.0) to estimate the α diversity of the gut microbiota using the Shannon diversity observed_spesies and phylogenetic diversity (PD_whole_tree) indices. The nonparametric Wilcoxon test was used to evaluate the significant differences between the gut microbial α diversity before and after the intervention. A nonparametric Wilcoxon test was performed to demonstrate the significant difference in the gut microbial β diversity pre- and post-intervention within and between the two groups using weighted and unweighted UniFrac distances determined using QIIME software (Version 1.7.0). Principal coordinate analysis (PCoA) was used to visualize the β diversity between the two groups before and after the intervention. In this trial, PCoA ordination plots were created based on operational taxonomic unit (OTU) abundance using Bray–Curtis (BC) distance. The vegan package’s permutational multivariate analysis of variance (PERMANOVA) function was used to examine the significance of the gut microbial β diversity before and after the intervention. Additionally, a *t*-test was utilized to determine species at various taxon ranks that varied significantly between the two periods. Statistical significance was defined as a *p*-value < 0.05. Further analysis was performed using linear discriminant analysis (LDA) effect size (LEfSe). By combining common statistical tests for statistical significance with additional tests conveying biological consistency and effect relevance, LEfSe ascertains the characteristics of an organism that are most likely to account for variations across interventional groups. The threshold on the logarithmic LDA score for discriminative features was set at 2.0, and the *α*-value for the Wilcoxon test was set at 0.05. All bioinformatic analyses were executed using the statistical and coding tool R (version 2.15.3).

#### 2.5.4. Functional Prediction Based on 16S rRNA Gene Data

To examine the potential metabolic function of microorganisms during the probiotic intervention, the representative sequences comprising the top 500 taxa were used to infer on Kyoto Encyclopedia of Genes and Genomes (KEGG) database using Phylogenetic Investigation of Communities’ Reconstruction of Unobserved States (PICRUSt2) pipeline (https://github.com/picrust/picrust2/, accessed on 1 May 2022) as previously described [43]. The gut microbial profiles of the probiotics and placebo groups before and at the end of intervention were investigated. To improve the accuracy and reliability of the KEGG pathways, a web-based tool, MicrobiomeAnalyst for comprehensive statistical, visual, and meta-analysis of microbiome data (https://www.microbiomeanalyst.ca/, accessed on 1 May 2022), was used to remove extremely low-abundance and low-variance KEEG orthologs (KO) and Clusters of Orthologous Groups (COG) from each sample, respectively [44]. The significant shift within the interventional group was analyzed using the Wilcoxon test in SPSS version 23 (SPSS, Chicago, IL, USA; *p*-value < 0.05).

### 2.6. Sample Size

The sample size calculation was based on Kijmanawat et al. [45]. A sample size of 66 participants per arm was sufficient for achieving a mean reduction of 3.67 mg/dL and a standard deviation of 7.44 mg/dL in the FBG levels, with 80% power and a two-sided 5% significance level. However, 83 participants were chosen per arm taking into account the 25% dropout rate and noncompliance.

### 2.7. Randomization and Blinding

A random allocation sequence with a 1:1 ratio was computer-generated. Simple randomization without stratification was used in this trial. The random allocation sequence participant enrollment and intervention assignment were created by qualified professionals who were not involved in the trial. The contents of the sachets were hidden from participants and researchers alike. Sachets had either “GDM-A” or “GDM-B” code, but the two groups’ content had the same look, fragrance, texture, and taste. The codes were not broken until after analyses were completed.

### 2.8. Statistical Analysis

This trial implemented a per-protocol (PP) analysis and included only the participants who had completed the intervention with good compliance (≥85%). All statistical analyses were conducted using SPSS software, version 23 (SPSS, Chicago, IL, USA). Variables were assessed for normal distribution using the Kolmogorov–Smirnov test as well as for skewness, and kurtosis. Continuous variables that were roughly normally distributed were shown as mean and standard deviation (SD) or 95% confidence interval (CI). Continuous variables that were not normally distributed were presented as median and interquartile ranges. Frequency (*n*) and percentage (%) were used to present categorical data. Baseline continuous variables were compared between the two groups using an independent sample *t*-test. A Mann–Whitney U test was used to compare baseline continuous variables that were not normally distributed. Categorical variables were tested using a Fisher’s exact test.

A paired *t*-test was used to compare dietary intake and physical activity before and after the study. General linear model (GLM) repeated-measures analysis of variance (ANOVA) was used to determine the mean differences (MD) between outcomes before and after the study. GLM repeated measures were analyzed independently for each group to obtain the MD of outcomes within each group. Meanwhile, choosing the intervention grouping (probiotics vs. placebo) as the between-subjects factor in the analysis of GLM repeated measures allowed for the determination of the MD in outcomes between the two groups. In order to limit the influence of baseline outcomes on post-intervention results, GLM univariate analysis of covariance (ANCOVA) was chosen with baseline outcome as a covariate. The effect sizes of the results were calculated using the eta squared formula. Effect sizes were classified as small if they ranged between 0.01 and 0.05, medium if between 0.06 and 0.13, and large if their sizes ≥ 0.14 [46].

On the other hand, throughout the 12-week intervention, Wilcoxon and Mann–Whitney U tests were used to assess the MD of continuous variables that were not normally distributed within and across the groups.

## 3. Results

### 3.1. Participant Recruitment and Flow

**Figure 1:**
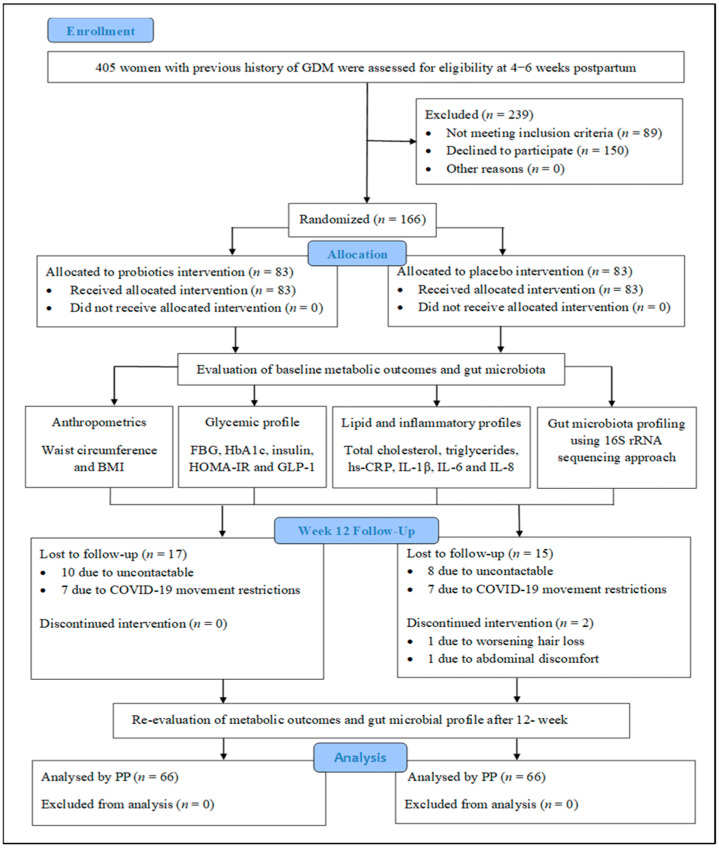
CONSORT diagram of randomized controlled trial. Abbreviations: GDM, gestational diabetes mellitus; BMI, body mass index; BG, fasting blood glucose; HOMA-IR, homeostasis model assessment of insulin resistance; GLP-1, glucagon like-peptide-1; hs-CRP, high sensitivity C-reactive protein; IL-1β, interleukin-1β; IL-6, interleukin-6; IL-8, interleukin-8; PP, per-protocol analysis.

Participant recruitment for this study was conducted between 1 March 2018 and 1 November 2020 due to the disruptions caused by the COVID-19 outbreak. The trial was completed on 15 April 2021. As shown in Figure 1, data related to 2350 women with a recent history of GDM were obtained from UKM Medical Center’s delivery records, and 405 post-GDM women were further assessed for eligibility. As only 166 of these post-GDM women were eligible and willing to participate in this trial, the sample was randomly segregated into the probiotics (*n* = 83) or the placebo (*n* = 83) group. The 12-week intervention was completed by 66 participants from each group, with 85–100% and 85–95% compliance rates in the probiotics and placebo groups, respectively. The majority (89.4%) of the probiotics group reported beneficial effects, such as a reduction in constipation and bloating, and 15% noted improvements in mood. Although 20% of the total sample (*n* = 34) was lost to follow-up mainly due to being uncontactable and to movement restrictions related to COVID-19, only 2 participants from the probiotics group discontinued supplementation due to side effects. None of the participants who completed the trial were on any medications or antibiotics, and none consumed foods or other supplements that were rich in prebiotics/probiotics/synbiotics during the 12-week intervention.

### 3.2. Baseline Characteristics of Participants

The baseline characteristics of all participants who completed the trial with good compliance (*n* = 132) are summarized in Table 1. With the exception of family history of T2DM, the sociodemographic, antepartum, and breastfeeding practices were comparable between the two groups. The age ranges of the participants in the probiotics and placebo groups were comparable (34.85 years ± 4.25, 34.27 years ± 4.79, respectively). Most participants had a pre-pregnancy BMI that would classify them as overweight/obese, were of Malay ethnicity, had completed tertiary education, and had three children. Based on the postpartum OGTT assessments, nearly half of the participants were categorized as GI and evenly distributed in the probiotics and placebo groups (30 vs. 31 participants, respectively). The majority of the participants started their intervention during four months postpartum. Dietary intake was consistent between the two groups, but total dietary fiber was higher in the probiotics group. Compared with Malaysian clinical practice guidelines, the intake of fat and cholesterol in both groups was higher, while their fiber intake was lower than recommended [39].

Participants from both groups reported similar levels of physical activity and the majority of this was related to work and household chores. Only a small amount came from leisure-time physical activities (40.89 min/week ± 48.61 for the probiotics group vs. 32.50 min/week ± 39.78 for the placebo group). The baseline assessments, which took place three to six months postpartum, revealed that participants in the probiotics group were found to be significantly more overweight/obese than those in the placebo group (97% vs. 83.3%, *p* = 0.016). Although both groups’ baseline FBG levels were within the normal range (<6.1 mmol/L), the HbA1c values were practically prediabetic (5.7%). Both groups’ hs-CRP values were elevated (>3.0 mg/L), indicating a high risk of inflammation [47]. In comparison with the placebo group, the probiotics group hs-CRP levels were significantly higher (5.05 mg/L ± 3.50 vs. 3.76 mg/L ± 3.03, *p* = 0.026, Table 1).

Table 2 displays the comparisons of 15 participants from each group’s subsets for baseline FSI, GLP-1, HOMA-IR, IL-1β, IL-6, and IL-8 levels. Their baseline biomarkers were comparable and within the normal range, as can be seen from the tabulated results, with the exception of their median HOMA-IR values, which were indicative of insulin resistance (>1.4) [48,49,50,51].

### 3.3. Changes in Dietary Habits and Physical Activity Levels after the 12-Week Intervention

The differences in food consumption and physical activity reported by the two groups are shown in Supplementary Table S1. At the conclusion of the trial, more types of foods were consumed by both groups. The differences were, however, not statistically significant. Conversely, both groups’ levels of overall physical activity levels dramatically decreased at the end of the trial (Supplementary Table S1).

### 3.4. Outcomes and Estimation

#### 3.4.1. Changes in Primary Outcomes

**Figure 2:**
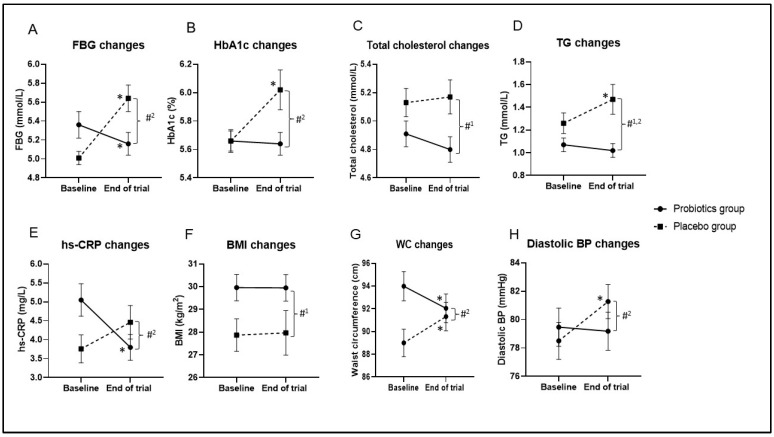
Changes in biochemical and anthropometric outcomes during 12-week intervention. Data are presented as mean ± standard error of the mean. (**A**) Fasting blood glucose (FBG) changes, (**B**) HbA1c changes, (**C**) total cholesterol changes, (**D**) triglyceride (TG) changes, (**E**) high-sensitivity C-reactive protein (hs-CRP) changes, (**F**) body mass index (BMI) changes, (**G**) waist circumference (WC) changes, (**H**) diastolic blood pressure (BP) changes. *p*-value < 0.05 was considered statistically significant. * Significant difference within the group. * *p*-value < 0.05 was considered statistically significant. #^1^ Significant difference between the two groups based on repeated-measures analysis of variance (ANOVA). #^2^ Significant interaction between the outcome and intervention group.

As shown in Figure 2, after the 12-week intervention, in the probiotics group, FBG levels were significantly lower than baseline following the 12-week intervention, with an MD of −0.20 mmol/L (95% CI = −0.36, −0.030; *p* = 0.0021; effect size = 0.079; Supplementary Table S2). However, in 20 people (31.7%) from the probiotics group, the FBG levels were slightly higher than the baseline. Conversely, FBG levels were significantly elevated in the placebo group, with MD of 0.64 mmol/L (95% CI = 0.32, 0.95; *p* < 0.001; effect size = 0.202; Figure 2; Supplementary Table S2). Using GLM repeated-measures ANOVA, no discernible difference in the FBG levels was detected between the two groups (MD = −0.06 mmol/L; 95% CI = −0.45, 0.33; *p* = 0.748; effect size = 0.001; Figure 2; Supplementary Table S3). However, after applying Greenhouse–Geisser correction, the interaction between the FBG and the intervention group was shown to be significant (*p* < 0.001; effect size = 0.145; Figure 2; Supplementary Table S3). Additional ANCOVA revealed that the probiotics and placebo groups’ FBG levels were significantly different from one another, with an MD of −0.81 mmol/L (95% CI = −1.16, −0.45; *p* < 0.001; effect size = 0.135; Supplementary Table S3) after adjusting for the baseline FBG levels.

The mean reduction in HbA1c was small in the probiotics group (MD = −0.02%; 95% CI = −0.11, 0.06; *p* = 0.585; effect size = 0.005; Figure 2; Supplementary Table S2), but the HbA1c levels were significantly higher in the placebo group, with a MD of 0.36% (95% CI = 0.18, 0.55; *p* < 0.001; effect size = 0.195; Figure 2; Supplementary Table S2) compared with the baseline. The interaction between HbA1c and intervention group was significant based on Greenhouse–Geisser correction (*p* < 0.001; effect size = 0.102; Figure 2; Supplementary Table S3). Additionally, HbA1c levels were significantly lower in the probiotics group compared with the placebo group, with a MD of −0.39% (95% CI = −0.59, −0.19; *p* < 0.001; effect size = 0.105; Supplementary Table S3) after adjusting for the baseline HbA1c levels.

**Figure 3:**
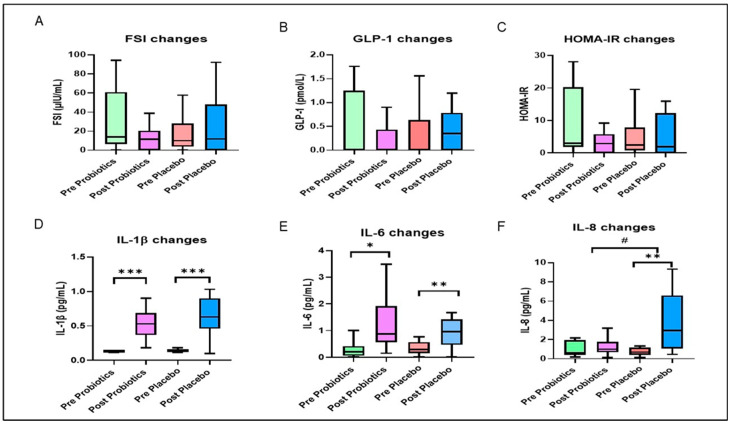
Changes in glycemic and inflammatory outcomes during the 12-week intervention. The box represents the interquartile range (IQR), the inside line represents the median, and the whisker represents the maximum and minimum value. (**A**) Fasting serum insulin (FSI) changes, (**B**) active glucagon-like peptide-1 (GLP-1) changes, (**C**) homeostasis model assessment of insulin resistance (HOMA-IR) changes, (**D**) interleukin-1β (IL-1 β changes), (**E**) interlukin-6 (IL-6) changes, (**F**) interleukin-8 (IL-8) changes. * Significant difference within the group. * *p*-value < 0.05, ** *p*-value < 0.01, and *** *p*-value < 0.001 were considered statistically significant. # Significant difference between the two groups. Changes in Inflammatory Outcomes.

There were no significant variations in pre- and post-intervention FSI, HOMA-IR, or GLP-1 within or between groups (*p* > 0.05; Figure 3; Supplementary Table S4). However, the median of FSI levels were slightly lower in the probiotics group at the end of the trial compared with the baseline level (13.99 μIU/mL [54.43] vs. 13.32 μIU/mL [34.33]; *p* > 0.05) but were elevated in the placebo group (13.16 μIU/mL [51.45] vs. 14.32 μIU/mL [66.21]; *p* > 0.05; Figure 3; Supplementary Table S4).

#### 3.4.2. Changes in Anthropometric Measurements Outcomes

As can be seen from Figure 2, BMI did not change significantly in either group during the 12-week intervention (*p* > 0.05; Supplementary Table S2) but differed significantly between the groups based on the GLM repeated-measures ANOVA (MD 2.04 kg/m^2^; 95% CI = 0.09, 4.00; *p* = 0.041; effect size = 0.032; Figure 2; Supplementary Table S3). The results reported in Supplementary Table S2 further indicate that the WC was significantly reduced in the probiotics group (MD = −1.93 cm; 95% CI = −3.01, −0.86; *p* < 0.001; effect size = 0.167; Figure 2) but significantly increased in the placebo group (MD = 2.32 cm; 95% CI = 1.15, 3.48; *p* < 0.001; effect size = 0.194; Figure 2). Significant interaction was noted between WC and the intervention group using Greenhouse–Geisser correction (*p* < 0.001; effect size = 0.140; Figure 2; Supplementary Table S3). This finding remained significant after adjusting for the baseline WC (MD = −3.82 cm; 95% CI = −5.42, −2.22; *p* = 0.006; effect size = 0.057; Supplementary Table S3). Conversely, pre- and post-intervention systolic blood pressure levels were comparable within and between the two groups (*p* > 0.05). For the diastolic pressure, the placebo group showed significant elevation after the intervention (MD = 2.77 mmHg; 95% CI = 0.61, 4.93; *p* = 0.013; effect size = 0.092; Figure 2; Supplementary Table S2), and the mean difference between the two groups was significant after adjusting for the baseline levels (MD = −2.74 mmHg; 95% CI = −5.31, −0.17; *p* = 0.037; effect size = 0.033; Figure 2; Supplementary Table S3).

#### 3.4.3. Changes in Lipid Outcomes

The mean total cholesterol level was significantly lower in the probiotics group compared with the placebo group based on GLM repeated-measures ANOVA (MD = −0.29 mmol/L; 95% CI = −0.55, −0.04; *p* = 0.026; effect size = 0.037; Figure 2; Supplementary Table S3). Mean triglycerides (TG) were significantly elevated in the placebo group after the 12-week intervention (MD = −0.21 mmol/L; 95% CI = 0.04, 0.39; *p* = 0.017; effect size = 0.085; Figure 2; Supplementary Table S2). The TG levels were significantly lower in the probiotics group compared with the placebo group based on GLM repeated-measures ANOVA (MD = −0.32 mmol/L; 95% CI = −0.55, −0.10; *p* = 0.006; effect size = 0.057; Figure 2; Supplementary Table S3).

After the 12-week intervention, as shown in Figure 2, the hs-CRP levels were significantly lower in the probiotics group (MD = −1.25 mg/L; 95% CI = −1.96, −0.54; *p* < 0.001; effect size = 0.160; Supplementary Table S2) and were higher in the placebo group (MD = 0.70 mg/L; 95% CI = −0.01, 1.40; *p* = 0.052; effect size = 0.057) compared with the baseline. The interaction between hs-CRP and the intervention group was significant based on Greenhouse–Geisser correction (*p* < 0.001; effect size = 0.104; Figure 2; Supplementary Table S3). Moreover, hs-CRP levels were significantly different between the two groups after adjusting for the baseline values (MD = −1.42 mg/L; 95% CI = −2.31, −0.52; *p* = 0.002; effect size = 0.071; Supplementary Table S3). After the trial, the IL-1β and IL-6 levels were significantly higher in both groups compared with the baseline (*p* < 0.05; Figure 3; Supplementary Table S4). The IL-8 levels were significantly higher at the end of the trial within the placebo group compared with the baseline (*p* = 0.006; Figure 3; Supplementary Table S4). In addition, IL-8 levels were significantly different between the two groups (*p* = 0.026; Figure 3; Supplementary Table S4).

#### 3.4.4. Changes in Relative Abundance of Gut Microbial Compositions

**Figure 4:**
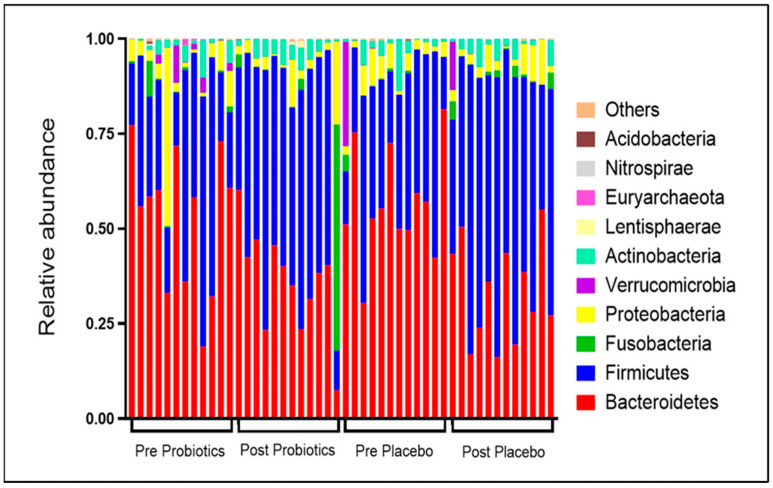
Changes in relative abundance of top 10 gut microbial compositions pre- and post-intervention. Each stacked bar resembles the top 10 gut microbial compositions at the phyla level of a participant.

At the phylum level, the baseline relative abundance of gut microbial compositions in both groups was dominated by Bacteroidetes followed by Firmicutes, Proteobacteria, and Verrucomicrobia (Figure 4; Supplementary Table S5). The pattern of baseline gut microbial composition within the probiotics group showed slight inter-individual variation; specifically, Proteobacteria and Firmicutes were more enriched than Bacteroidetes in a small number of participants. In comparison, the pattern of baseline gut microbial composition was uniformly dominated by Bacteroidetes in the placebo group. After the 12-week intervention, the relative abundance of Bacteroidetes was depleted and Firmicutes was elevated (Figure 4) compared with the baseline. The ratio of Bacteroidetes to Firmicutes was significantly lower within the two groups at the end of the trial when compared with the baseline (*p* < 0.05).

#### 3.4.5. Changes in Gut Microbial α Diversity

**Figure 5:**
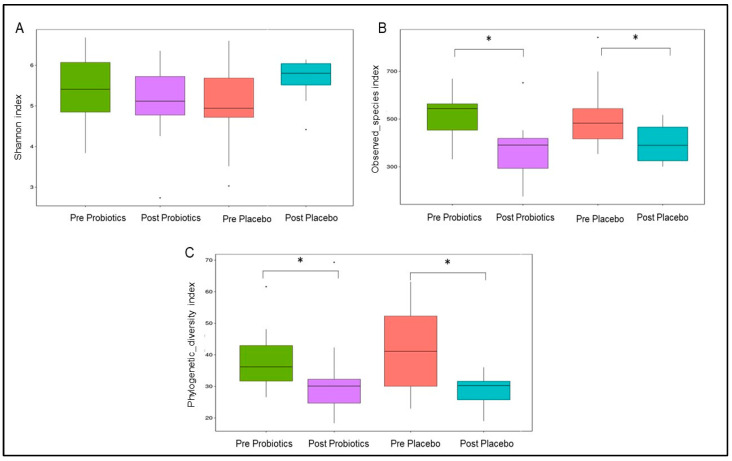
Changes in gut microbial α diversity within the two groups. (**A**) Shannon diversity index represents gut microbial evenness. (**B**) Observed_species index represents gut microbial richness. (**C**) Phylogenetic_diversity index represents gut microbial phylogenetic difference. * *p*-value < 0.05 was considered as statistically significant.

The Shannon diversity index reflects gut microbial evenness, and the index was stable during the trial in both groups (*p* > 0.05; Figure 5A). In terms of microbial richness, the observed species index indicated significant reductions in the gut microbial composition in the intervention group (MD = −15.83; 95% CI = −26.12, −5.55; *p* = 0.0031; Figure 5) and the placebo group (MD = −11.75; 95% CI = −22.04, −1.46; *p* =0.026; Figure 5) compared with the baseline. The gut microbial phylogenetic diversity was also significantly lower within the placebo group (MD = −13.42; 95% CI = −24.08, −2.75; *p* = 0.015; Figure 5C) and the probiotics group (MD = −11.08; 95% CI = −21.75, −0.42; *p* = 0.042; Figure 5C) compared with the baseline. Nonetheless, between the two groups, the Shannon diversity, observed species, and phylogenetic diversity indices were comparable (*p* > 0.05; Figure 5). 

#### 3.4.6. Changes in Gut Microbial β Diversity

**Figure 6:**
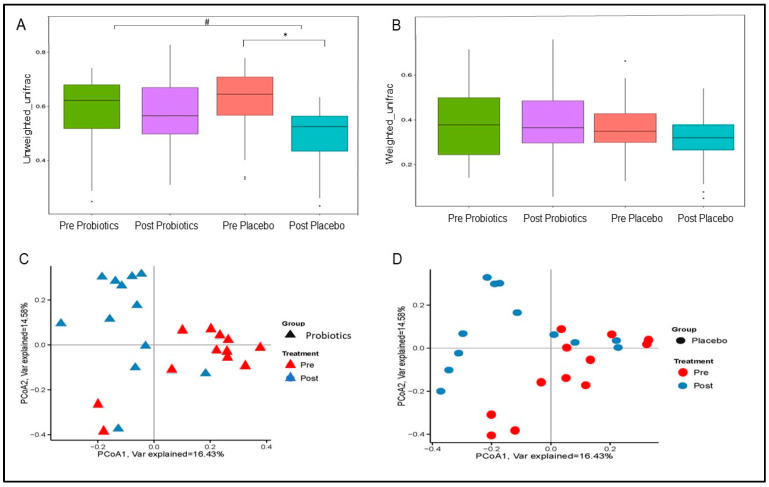
Changes in gut microbial β diversity within the two groups. (**A**) Boxplots of unweighted UniFrac distance. (**B**) Boxplots of weighted UniFrac distance. Principal coordinate analysis (PCoA) plots based on Bray–Curtis (BC) distance within the probiotics (**C**) and placebo (**D**) groups. *p*-value < 0.05 was considered statistically significant. * Significant difference within the group. # Significant difference between the two groups.

Unweighted UF distance within the placebo group was significantly lower at the end of the trial (MD = −86.27; 95% CI = −110.24, −62.30; *p* < 0.001; Figure 6A) and differed significantly from the probiotics group (MD = −49.91; 95% CI = −73.88, −25.94; *p* < 0.001; Figure 6A). The placebo group also showed reduction in the weighted UF distance (MD = −25.15; 95% CI = −50.98, 0.68; *p* = 0.056; Figure 6B) compared with the baseline. Meanwhile, the weighted UF distance of the probiotics group at the end of the trial was not significantly changed from the baseline (MD = 5.79; 95% CI = −20.04, −31.62; *p* = 0.6594; Figure 6B). PCoA based on BC distance revealed that the post-intervention gut microbial communities (β diversity) within both groups were different from the pre-intervention gut microbial communities (Figure 6C,D). Changes in gut microbial communities within the probiotics groups showed more clear separation and clustering compared with the placebo group, which was consistent with the PERMANOVA results (*p* = 0.002 vs. *p* = 0.004; Figure 6C,D). However, PERMANOVA did not reveal a significant difference in the post-intervention gut microbial β diversity between the two groups (*p* > 0.05).

**Figure 7:**
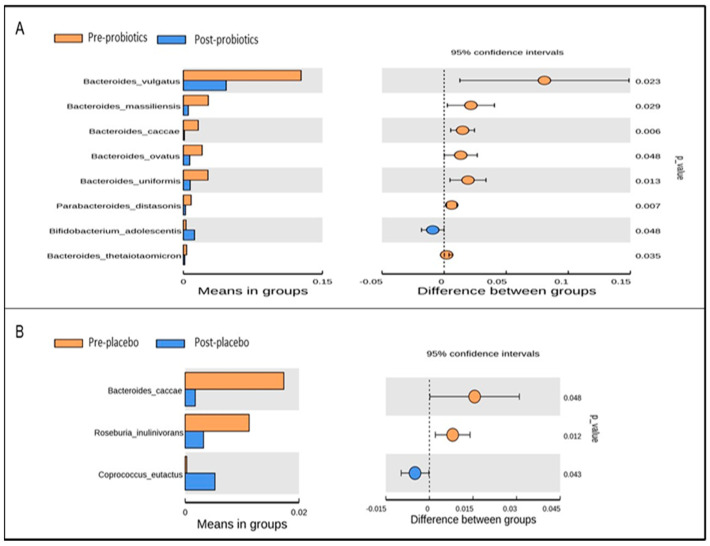
Significantly abundant gut microbial taxa at the species level within the probiotics (**A**) and placebo (**B**) groups pre- and post-intervention, identified using *t*-test analysis. *p*-value < 0.05 was considered statistically significant.

At the species level, *Bifidobacterium adolescentis* was significantly elevated and gut species such as *Bacteroides vulgatus*, *Bacteroides massiliensis*, *Bacteroides uniformis*, and *Parabacteroides distasonis* were significantly depleted in the probiotics group at the end of the trial based on *t*-test analysis (Figure 7A). However, only *Bacteroides caccae* and *Roseburia inulivorans* were reduced and *Coprococcus eutactus* was increased in the placebo group at the end of the 12-week intervention based on *t*-test analysis (Figure 7B).

**Figure 8:**
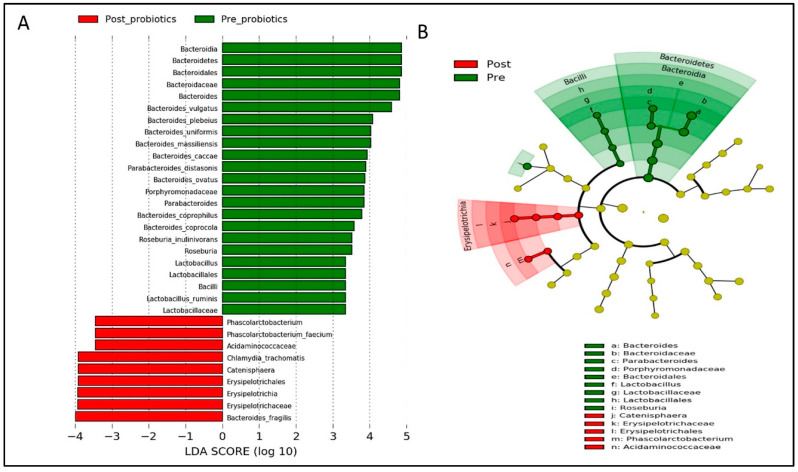
Linear discriminant analysis (LDA) effect size (LEfSe) analysis showing significantly abundant gut microbial taxa down to species level within the probiotics group. (**A**) Histogram represents the LDA scores for differentially abundant gut microbial taxa within the probiotics group, pre- and post-intervention. (**B**) Cladogram represents the taxonomic structure and relative abundance of the predominant gut microbial taxa within the probiotics group, pre- and post-intervention. The size of each dot in the cladogram represents the relative abundance of the identified gut microbial taxa. α < 0.05 and LDA score ≥ 2.0 were considered statistically significant.

**Figure 9:**
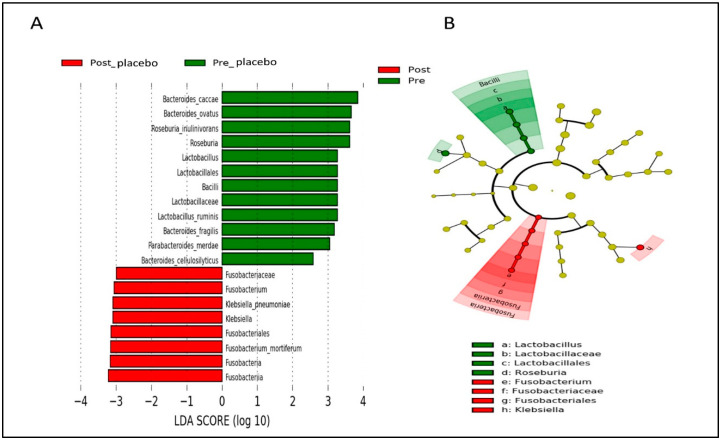
Linear discriminant analysis (LDA) effect size (LEfSe) analysis showing significantly abundant gut microbial taxa down to species level within the placebo group. (**A**) Histogram represents the LDA scores for differentially abundant gut microbial taxa within the placebo group, pre- and post-intervention. (**B**) Cladogram represents the taxonomic structure and relative abundance of the predominant gut microbial taxa within the placebo group, pre- and post-intervention. The size of each dot in the cladogram represents the relative abundance of the identified gut microbial taxa. α < 0.05 and LDA score ≥ 2.0 were considered statistically significant.

Based on LEfSe analysis, several gut microbial taxa (i.e., Bacteroidetes, *Parabacteroidetes distasonis*, *Roseburia*, and *Lactobacillus*) were significantly abundant in both groups at baseline (Figure 8 and Figure 9). Post-intervention, gut microbial taxa such as *Bacteroides fragilis*, *Erysipelotrichia*, and *Phascolarctobacterium* were increased in the probiotics group (Figure 8), while gut microbial taxa derived from Fusobacteria and *Klebsiella* were enriched in the placebo group (Figure 9).

Additionally, the protein-coding genes of the most abundant gut microbial taxa (top 500) were annotated using the KO and COG databases to predict gut microbial functions and metabolic pathways (Supplementary Figure S1; Tables S6 and S7). After completing the 12-week intervention, both groups showed a significant reduction in carbohydrate transport and metabolism pathway: a higher reduction in COG annotation was noted for the probiotics group (*p* = 0.008 vs. *p* = 0.034; Supplementary Figure S1A) compared with the baseline. Moreover, the secondary metabolite biosynthesis transport and catabolism pathway was significantly increased in the probiotics group after the 12-week intervention (*p* = 0.015). The analysis of gut microbial function and metabolic pathways using KO further showed a significant reduction in the lipid metabolism (*p* = 0.003; Supplementary Figure S1B), glycan biosynthesis and metabolism (*p* = 0.008), carbohydrate metabolism (*p*= 0.003; Supplementary Figure S1B), and amino acid metabolism pathways (*p* = 0.003; Supplementary Figure S1B) within the probiotics group. The lipid metabolism (*p* = 0.034), glycan biosynthesis, and metabolism pathways (*p* = 0.028) were also significantly reduced within the placebo group but to a lesser extent than the probiotic group (Supplementary Figure S1B). In comparison to the baseline, although not statistically significant, the energy metabolism pathway was elevated in the probiotics group (*p* = 0.084).

As previously noted, only two participants from the probiotics group discontinued the intervention and left the study due to worsening hair loss (*n* = 1) and acute abdominal discomfort (*n* = 1). A small number of participants from the probiotics group complained of mild side effects such as bloating (*n* = 1), light headache (*n* = 1), increased appetite (*n* = 2), and increased frequency of passing motion in breastfed infants (*n* = 3), but they did not discontinue supplementation; these side effects were temporary and resolved gradually after continuous probiotic supplementation.

## 4. Discussion

Probiotics supplementation’s positive effects on the metabolic and inflammatory outcomes in postpartum women with a recent history of GDM are highlighted in this innovative randomized clinical trial. Our analyses showed that probiotics supplementation for 12 weeks significantly decreased FBG, waist circumference, and hs-CRP in post-GDM women. Despite the probiotics group’s minimal changes from baseline in HbA1c, total cholesterol, TG, and IL-8 levels, these outcomes were significantly lower in the probiotics group after the 12-week intervention when compared with the placebo group. Moreover, taking probiotics helped to restore gut microbial profiles, gut microbial functions, and metabolic pathways without having a negative impact on health.

Recent evidence showed that the risk of developing T2DM increases 10-fold for women with a previous history of GDM compared with healthy women. Probiotics supplementation is thus beneficial in this cohort, as it can improve glucose metabolism [32]. In the present study, we identified that the FBG levels were significantly lower in the probiotics group, by 0.20 mmol/L, compared with the baseline. However, the effect size was at the low end of moderate (0.08), and 31.7% of the participants from the probiotics group did not show improvement. Probiotics might have modest effects on FBG levels because the baseline FBG levels in asymptomatic post-GDM women were within the normal range. Thus, it is less likely that the probiotics would reduce the FBG levels abruptly, and instead, the levels were maintained. This finding could also have been due to preexisting variations in the gut microbial composition, lifestyle changes during the COVID-19 outbreak, or other unobserved factors.

The baseline HbA1c levels in both groups were near prediabetic status, and probiotic supplementation reduced the HbA1c levels minimally in post-GDM women, by 0.02%, compared with the baseline. Although the effects of the probiotics on overall glycemic profiles are limited, it should be noted that even a small reduction in the glycemic profile is beneficial to reduce the progression of T2DM among post-GDM women [52]. A subgroup analysis by Firouzi et al. [28] reported that probiotics were less efficient in overweight/obese participants. Therefore, the effects of probiotics on HbA1c levels might have been small because the majority of our participants were either obese or overweight. Our participants consumed very low dietary fiber intake throughout the trial because they were instructed to maintain similar dietary intakes and physical activities to avoid confounding factors. This might have contributed to the inefficiency of the probiotics supplementation because vegetables and fruits are important source of prebiotics that promote the modulation of gut microbiota [37]. This postulation is consistent with Laitinen et al. [53], who found significant improvement in glycemic outcomes during pregnancy that persisted during the postpartum period after probiotic supplementation was given together with dietary counseling. Moreover, previous trials that involved adults with T2DM may have shown greater improvement in HbA1c levels than in our trial because the consumption of hypoglycemic agents may have confounded the results [28].

Postpartum weight retention and obesity are important factors that determine postpartum GI [54]. Authors of most studies involving pregnant women with GDM did not find significant improvement in weight gain and BMI after probiotic supplementation [55,56,57]. In contrast, Dolatkhah et al. [58] noted that the weight gain was significantly lower in the probiotics group, concurring with our results indicating significantly reduced WC in the probiotics group. However, we found that probiotics were not efficient enough to reduce BMI in the post-GDM women, in line with the results reported by other authors [45,59]. Due to the COVID-19 outbreak, it is likely that the dietary intake and physical activity levels were modified in our cohort, which might have influenced our results. In addition, a longer duration of probiotics supplementation may be required (≥12 weeks) to reverse certain metabolic outcomes as the effects may vary according to the probiotic’s strains, doses, duration, age, health status, and intestinal transit time [30,32].

On the other hand, probiotic supplementation was positively associated with lipid metabolism. It might be mediated through the modulation of SCFAs and peroxisome proliferator-activated receptor (PPAR)-γ gene; these mediators balance the host energy expenditure and lipid storage capacity [22,55]. According to the available evidence, probiotic supplementation can significantly reduce total cholesterol [60], very low-density lipoprotein, and TG levels in pregnant women with GDM [55,56,59]. Findings yielded by most studies involving adults with T2DM supported the beneficial role of probiotics in lipid metabolism but are inconsistent [61]. In this study, the lipid parameters (i.e., total cholesterol and TG) levels were stable in the probiotics group, and the TG levels increased significantly in the placebo group. The changes might not have been significant because the baseline values were already within the normal range. Second, the effect size in our study may have been small because the probiotics were found to affect the lipid profiles in a dose-dependent and duration-dependent manner [62].

In the present study, hs-CRP levels were significantly high at the baseline, and this finding supported the association between GDM, obesity, and inflammation. Post-intervention, the hs-CRP levels were significantly reduced among post-GDM women in the probiotics group, which is consistent with previous studies [57,63,64]. Extant evidence further indicates that probiotic supplementation might have regulated the inflammatory response by strengthening the gut epithelial permeability, reducing metabolic endotoxemia, and maintaining the inflammatory markers within the normal range [22]. In addition to hs-CRP, two previous studies involving pregnant women with GDM showed that IL-6 and tumor necrosis factor-α (TNF-α) levels were reduced in the probiotics group [63,64]. In contrast, we noted that IL-6 and IL-1β levels were significantly increased in both groups after a 12-week intervention. The elevation of IL-6 and IL-1β levels may be due to postpartum changes in post-GSM women and not related to the probiotic supplementation [65]. For instance, serum IL-6 concentration can be elevated in pregnant women with GDM and remain elevated at 2 months postpartum [65]. IL-6 is also positively correlated with hyperglycemia and insulin resistance/sensitivity indices [65]. The IL-8 levels, however, remained normal among the post-GDM women in the probiotics group, and their values were significantly lower than those measured in the placebo group. Our results are supported by the findings reported by other authors that the oral administration of *Lactobacillus rhamnosus* CCFM0528 to diabetic mice for 12 weeks reduced the concentration of IL-8 [66]. Conversely, Babadi et al. [55] did not find significant changes in IL-8 gene expression among pregnant women with GDM after probiotic supplementation. The roles of probiotics in immunomodulation among post-GDM women are still uncertain and should be interpreted carefully because the sample size to evaluate the interleukins was small.

Currently, extensive attention is focused on elucidating the role of gut microbiota in host metabolism. For example, during the postpartum period, Fugmann et al. [19] found that the gut microbial composition in post-GDM women had a more Firmicutes and less Bacteroidetes than the control group. The findings related to gut microbial dysbiosis obtained in this trial were similar to those reported by Fugmann et al. [19] and are comparable with the gut microbial dysbiosis of adults with T2DM [20,67]. Probiotic supplementation has been shown to modulate gut microbial profiles and improve glycemic control [68,69]. Post-intervention, we found a significantly reduced ratio of Bacteroidetes to Firmicutes in both groups. The administration of multi-strain probiotics in obese pregnant women with GDM has been shown to modulate the gut microbial profile by improving the gut microbial α diversity [70,71]. Halkjaer et al. [71], however, found that probiotic supplementation led to no changes in the gut microbial β diversity of obese pregnant women. In comparison, we found a reduction in the gut microbial α diversity in the probiotics group, while the gut microbial β diversity was maintained and demonstrated better clustering compared with the placebo group. The gut microbial diversity may not have significantly increased in our post-GDM women because most participants had inadequate fiber intake, which is essential for gut microbial colonization, and obesity has been associated with low gut microbiota richness [72].

It is also noteworthy that beneficial gut microbiota such as Lachnospiraceace, *Dubosiealla*, *Bifidobacterium*, *Lactobacillus*, *Olsenella*, *Allobaculum*, and *Clostridium sensu stricto* are typically elevated following probiotic supplementation [68,69,70]. Our findings are in agreement with those reported in other studies, as *Bifidobacterium adolescentis*, *Erysipelotrichia*, and *Phascolarcbacterium* were enriched among post-GDM women after probiotic supplementation. Increases in these beneficial bacteria derived from Firmicutes may mediate host metabolism through the regulation of SCFA production [22]. Additionally, we observed that several Gram-negative bacteria derived from Bacteroidetes (i.e., *Bacteroides vulgatus*) were markedly depleted in the probiotics group, which might have reduced the LPS levels, suppressed metabolic endotoxemia, attenuated inflammatory responses, and improved insulin signaling in post-GDM women [22,73]. Our finding indicates that probiotic supplementation with low dietary fiber intake is still able to colonize and modulate the gut microbiota but might be inefficient for increasing α diversity within only 12 weeks.

Ferrocino et al. [18] predicted gut microbial function and metabolic pathway using KO databases and discovered enrichment of several metabolic pathways such as glycolysis/gluconeogenesis (KO00010), fructose and mannose metabolism (KO00051), galactose metabolism (KO00052), starch and sucrose metabolism (KO005009), and biosynthesis of amino acids (KO01230) in pregnant women with GDM. In adults with T2DM, glycolysis/gluconeogenesis pathways have been found to be more abundant than in controls, and these findings are associated with sucrose intake [74]. However, the other glycan degradation pathway is found to be less abundant in adults with T2DM, and this phenomenon is associated with pancreatic beta cell function [74,75]. Therefore, the modulation of these pathways using probiotics may be beneficial in post-GDM women. Zheng et al. [70] found that the carbohydrate and membrane transport pathways were less abundant among pregnant women with GDM who were given probiotics. The authors postulated that probiotic supplementation regulates glucose homeostasis by inhibiting the carbohydrate and membrane transport pathways. Our findings are similar to those reported by Zheng et al. [70]. Administration of *Bacillus coagulans* LBSC in adults with irritable bowel syndrome has been shown to influence their gut microbial profiles and enhance several metabolic pathways, such as energy metabolism and biosynthesis of glycan [76]. We also observed energy metabolism enrichment in the probiotics group, which would in turn promote energy expenditure and maintain the metabolic outcomes in post-GDM women. However, the glycan biosynthesis and metabolism pathways were significantly reduced among the post-GDM in the probiotics group; as *Bacteroides* is linked to glycan metabolism [77,78], this may be a possible reason why. Interestingly, probiotics supplementation significantly increased the secondary metabolite biosynthesis transport and catabolism pathway in post-GDM women. Increasing secondary metabolites has been postulated to improve glucose homeostasis in adults with T2DM [79]. Nonetheless, the associations between the aforementioned pathways and metabolic outcomes in post-GDM women remain inconclusive. Thus, further studies are warranted to determine their potential roles in regulating metabolic outcomes in post-GDM women.

This is the first 12-week randomized, double-blind, placebo-controlled, parallel-group clinical trial as a part of which the role of probiotics in women with a recent history of GDM during the postpartum period was evaluated. All previous trials focused on pregnant women with GDM and were conducted for shorter periods (8 weeks and below) [32]. As mentioned above, we did not observe significant changes or large effect sizes in certain outcomes, and several noteworthy limitations may have influenced the outcomes of our trial. The overall sample size was adequate, but our evaluation of certain parameters such as FSI and gut microbial profile was based on smaller samples (30 and 24 individuals, respectively). Consequently, it was not possible to observe significant changes in the probiotics group after the 12-week intervention. Second, this trial was conducted at a single health center, and the sample composition was not representative of the Malaysian population.

More importantly, even though the response to probiotic supplementation depends on the preexisting gut microbial composition and lifestyle changes, these factors were not considered in our analyses [32]. For example, individuals with Bacteroides predominance responded well to bifidobacterial-increasing intervention, whereas individuals in whom Prevotella was predominant responded well to a high-fiber diet intervention rich in arabinoxylans and β-glucans to improve weight loss [18]. Separately, Kong et al. [69] found that mice fed a high-sucrose diet responded better to probiotic supplementation than the mice fed a high-fat diet. It is also worth noting that due to the COVID-19 pandemic, most of our participants continued the same unhealthy dietary and exercise patterns and some even had poorer lifestyles than prior to the COVID-19 outbreak. Thus, conducting studies on participants with suitable preexisting gut microbial compositions and healthy lifestyles is essential. In this study, the participants in the probiotic groups had significantly higher BMI and fiber intake compared with the placebo group. This might have influenced the results. Future researchers are recommended to employ block randomization with stratification (i.e., BMI, age, dietary intake, and physical activity) to ensure equal baseline characteristics.

Moreover, as certain baseline biochemical markers that were tested at 3–6 months postpartum were within the normal range, further research focusing on participants with deranged biochemical profiles is warranted to elicit significant changes. For GLP-1, it is recommended to examine both active and total GLP-1, and sampling should be performed at a few intervals (fasting and a few intervals after meals) to assess the role of probiotic supplementation on GLP-1 levels [80]. Finally, LPS, SCFA and PPAR-γ levels should be assessed to verify the links between these markers and probiotics in post-GDM women. Additionally, we were unable to confirm the presence of the probiotic strains in the intestine because we only employed 16S rRNA gene sequencing.

## 5. Conclusions

Low-grade inflammation is linked to persistent postpartum obesity and GI in women with a previous history of GDM and is a major public health concern. In this trial, probiotics supplementation improved metabolic outcomes, gut microbial profiles, and certain inflammatory markers in postpartum women with a recent history of GDM. The advantages and negative effects of probiotics in post-GDM women should be carefully understood and customized, as these effects may vary between individuals depending on lifestyle and preexisting gut bacteria makeup. Given that the probiotics’ overall beneficial effects on metabolic outcomes were minimal, we deduced that their activities in this trial were geared more toward reducing illness severity than treating it. In practice, a combination of a healthy lifestyle and probiotics supplementation should be adopted as a viable preventive therapy in order to remodel gut dysbiosis and metabolic outcomes in post-GDM women. Further studies incorporating a wide participant pool from several centers are also required in order to better determine the functions of probiotics in post-GDM women.

## Figures and Tables

**Table 1:** Baseline characteristics of the participants categorized into probiotics and placebo groups.

Characteristics	Probiotics (*n* = 66)	Placebo (*n* = 66)	*p*-Value
Age (years)	34.85 ± 4.25	34.27 ± 4.79	0.487
Pre-pregnancy BMI (kg/m^2^)	28.56 ± 5.10	27.12 ± 5.52	0.122
Ethnicity, *n* (%)			1.000 ^#^
Malay	60 (90.9)	60 (90.9)	
Non-Malay	6 (9.1)	6 (9.1)	
Education level, *n* (%)			0.059 ^#^
Secondary	15 (22.7)	26 (39.4)	
Tertiary	51 (77.3)	40 (60.6)	
Number of children	3 ± 1	3 ± 1	0.813
Family history of T2DM, *n* (%)	52 (78.8)	40 (60.6)	0.037 ^#^*
Received hypoglycemic agents in pregnancy, n (%)	29 (43.9)	26 (39.4)	0.724 ^#^
Exclusive breastfeeding, *n* (%)	30 (45.5)	25 (37.9)	0.480 ^#^
**Postpartum OGTT assessment,** *n* (%)			
Frequency of postpartum GI, *n* (%)	30 (45.5)	31 (47.0)	1.000 ^#^
**Pre-intervention assessment**			
Months after delivery	4.08 ± 1.09	4.12 ± 1.07	0.809
**Macronutrient intake**			
Total energy intakes (kcal/day)	1600.82 ± 341.31	1606.88 ± 319.96	0.916
Carbohydrate (% total kcal)	58.2 ± 7.9	56.0 ± 6.9	0.095
Fat (% total kcal)	33.9 ± 4.7	32.3 ± 13.1	0.056
Proteins (% total kcal)	16.8 ± 2.4	16.1 ± 2.5	0.116
Cholesterol intakes (mg/day)	224.05 ± 106.15	215.46 ± 125.40	0.672
Total dietary fiber (g/day)	6.45 ± 4.29	4.65 ± 2.27	0.003 **
**Physical activities (minutes/week)**			
Commuting	56.62 ± 49.87	62.05 ± 55.30	0.555
Leisure-time activities	40.89 ± 48.61	32.50 ± 39.78	0.286
Household activities	1491.71 ± 559.01	1520.99 ± 672.38	0.786
Activities at work	1470.98 ± 823.99	1230.68 ± 935.24	0.120
Total average 1-week physical activities	3060.21 ± 640.84	2846.21 ± 709.51	0.071
**Anthropometric measurements**			
BMI (kg/m^2^)	29.96 ± 4.67	27.87 ± 5.79	0.024 *
Waist circumference (cm)	93.99 ± 10.39	89.01 ± 9.78	0.005 *
Overweight/Obese, *n* (%)	64 (97.0)	55 (83.3)	0.016 ^#^*
Systolic blood pressure (mmHg)	115.74 ± 11.77	116.41 ± 12.77	0.756
Diastolic blood pressure (mmHg)	79.47 ± 10.97	78.50 ± 10.48	0.605
**Biochemical outcomes**			
FBG (mmol/L)	5.36 ± 1.14	5.00 ± 0.95	0.056
HbAIc (%)	5.66 ± 0.66	5.66 ± 0.56	0.944
Total cholesterol (mmol/L)	4.91 ± 0.70	5.12 ± 0.85	0.103
Triglycerides (mmol/L)	1.07 ± 0.77	1.26 ± 0.75	0.082
hs-CRP (mg/L)	5.05 ± 3.50	3.76 ± 3.03	0.026 *

Data are presented as mean ± standard deviation or as *n* (%). *p*-value was calculated using *t*-test for continuous variables, and ^#^ Fisher’s Exact test was used for categorical variables. * *p*-value < 0.05, ** *p*-value < 0.01 were considered statistically significant. Abbreviations: BMI, body mass index; OGTT, oral glucose tolerance test; GI, glucose intolerance; FBG, fasting blood glucose; hs-CRP, high-sensitivity C-reactive protein.

**Table 2:** Baseline glycemic and inflammatory biomarkers of the participants (*n* = 30).

Biomarkers	Probiotics (*n* = 15)	Placebo (*n* = 15)	*p*-Value
Fasting serum insulin (μIU/mL)	13.99 (54.43)	13.16 (51.45)	0.852
HOMA-IR	3.05 (18.37)	3.74 (14.45)	0.852
Fasting active GLP-1 (pmol/L)	0.00 (1.25)	0.00 (0.93)	0.909
Interleukin-1β (pg/mL)	0.13 (0.01)	0.13 (0.04)	0.868
Interleukin-6 (pg/mL)	0.26 (0.53)	0.29 (0.43)	0.820
Interleukin-8 (pg/mL)	0.65 (1.54)	0.80 (0.77)	0.852

Data are presented as median (interquartile range). The differences between the two groups were tested using a Mann–Whitney U test. Abbreviations: HOMA-IR, homeostasis model assessment of insulin resistance; GLP, glucagon-like peptide.

## Data Availability

Raw sequencing reads for all samples described in this project have been deposited in the NCBI Sequence Read Archive under accession no. PRJNA717644 (https://www.ncbi.nlm.nih.gov/sra/PRJNA717644).

## Supplementary Materials

The following supporting information can be downloaded at: https://www.mdpi.com/article/10.3390/nu14183878/s1, Table S1: Changes in dietary intake and physical activities of the participants during the 12-week intervention (*n* = 132), Table S2: Changes in biochemical and anthropometric outcomes among the participants during the 12-week intervention within the probiotics (n = 66) and placebo (*n* = 66) groups, Table S3: Changes in biochemical and anthropometric outcomes during the 12-week intervention between the probiotics (*n* = 66) and placebo (*n* = 66) groups, Table S4: Changes in glycemic and inflammatory biomarkers of the participants during the 12-week intervention within and between the probiotics (*n* = 15) and placebo (*n* =15) groups, Table S5: Relative abundance of top 10 gut microbial phyla pre- and post-intervention, Figure S1: Predictions of gut microbial functions and metabolic pathways pre- and post-intervention. (A) Annotated in COG database; (B) Annotated in KO database. Table S6: Predictions of gut microbial functions pre- and post-intervention annotated in the Clusters of Orthologous Group (COG) database, Table S7: Predictions of gut microbial metabolic pathways pre- and post-intervention annotated in the Kyoto Encyclopedia of Genes and Genomes (KEGG) Orthologs (O) database.
